# Left Ventricular Pacing In Patients With Congestive Heart Failure

**Published:** 2006-01-01

**Authors:** Yves Etienne, Marjaneh Fatemi, Jean-Jacques Blanc

**Affiliations:** Cardiology department, Brest University Hospital, France

**Keywords:** cardiac resynchronisation, left ventricular pacing, heart failure

## Abstract

Cardiac resynchronisation therapy (CRT) using biventricular (BIV) pacing has proved its effectiveness to correct myocardial asynchrony and improve clinical status of patients with severe congestive heart failure (CHF) and widened QRS. Despite a different effect on left ventricular electrical dispersion, left univentricular (LV) pacing is able to achieve the same mechanical synchronisation as BIV pacing in experimental studies and in humans. This results in clinical benefits of LV pacing at mid-term follow-up, with significant improvement in functional class, quality of life and exercise tolerance at the same extent as those observed with BIV stimulation in non randomised studies. Furthermore these benefits are obtained at lesser costs and with conventional dual-chamber devices. However, LV pacing has to be compared to BIV pacing in randomised trials before being definitely considered as a cost-effective alternative to BIV pacing.

## Introduction

Despite advances in drug treatment, congestive heart failure (CHF) remains a major health care problem associated with a poor quality of life and a high mortality rate. During the past decade, cardiac resynchronisation therapy (CRT) using biventricular (BIV) pacing emerged as a promising technique improving quality of life, exercise tolerance and mortality [1-3], and is now an admitted therapy in a selected population of patients with widened QRS and severe CHF despite optimal pharmacological therapy [4,5]. It has been well documented that intraventricular conduction disturbances, namely left bundle branch block, induce left  ventricular (LV) activation delay leading to discoordinated myocardial contraction and,  consecutively, to deleterious effects on myocardial systolic function, diastolic filling time and mitral regurgitation [6]. Finally this dyssynchronised LV function exaggerates clinical symptoms of CHF. The rationale for CRT is based upon the hypothesis that, by correcting inter and intra-ventricular asynchrony, BIV pacing improves LV function and favourably affects clinical condition and prognosis of patients with severe CHF and prolonged QRS. However it has never been demonstrated  that electrical resynchronisation induced by BIV pacing is a sine qua non condition to achieve better mechanical coordination as, in experimental data, and despite electrical dispersion, LV pacing does as well as BIV pacing on mechanical synchronisation with similar hemodynamic benefit [7].

Many other arguments support the concept that LV univentricular pacing by reversing intraventricular dysynchrony is sufficient in humans to improve LV function and clinical status at the same extent and at a lower cost-effective ratio than BIV pacing and this is in accordance with the fact that intraventricular asynchrony seems more relevant that interventricular asynchrony to predict prognosis in patients with dilated cardiomyopathy [8].

## Hemodynamic effects of pacing

The first clinical series with CRT were performed empirically using BIV pacing [9]. However, since this period , several studies in humans have reported similar hemodynamic benefit using either LV or BIV pacing during acute studies, showing a decrease in pulmonary pressures, an increase in cardiac outpout, systemic blood pressure, and dP/dt when compared to baseline or to right ventricular pacing in patients with severe CHF and  intraventricular conduction delays [10-12];. Some authors reported that LV stimulation had even a greater effect on LV dP/dt than BIV [11,12]. This hemodynamic benefit was independent of atrio-ventricular conduction as the same effects were observed in patients in sinus rhythm or atrial fibrillation [13]. Furthermore, improvement in myocardial contractility induced by LV pacing was obtained with the same extent of reduced energetic cost for the myocardium than with BIV pacing [14]. Finally, a similar reduction in mitral regurgitation was observed by echocardiography using the two pacing modes with a significant decrease by 30 to 50 % in mitral jet area or mitral regurgitant orifice area in non-randomised studies [15-17], despite less striking effects upon LV reverse remodeling during LV versus BIV pacing in some studies [15-17].

## Clinical benefits

Whether or not these hemodynamic benefits result in sustained improvement of clinical status during permanent LV stimulation has not been extensively investigated until now. However, some studies reported significant improvement in both exercise tolerance and quality of life at mid-term  follow-up during chronic LV pacing [18,19] or failed to demonstrate any superiority of BIV  pacing over LV pacing on clinical data[17,20]. Aurricchio et al compared 3 months of active LV pacing to 3 months of inactive pacing in 86 patients with severe CHF and wide QRS duration. They observed a significant increase in peak V0_2_ (2.46 ml/mn/kg), in 6 minute walk distance (47 m) and a decrease in quality of life score (8.1) during the active pacing period in the group of patients with the longest  QRS duration (above 150 ms) [18]. In another study comparing 2 active periods (4 weeks each) of uniLV and BIV pacing to baseline in a cross-over design, the same authors reported similar improvement in functional class, quality of life score, and exercise tolerance in the 2 pacing modes [20]. Our personal studies lead to the same conclusions: by comparing to baseline, we found after 12 months of LV pacing in 22 patients with severe CHF, sinus rhythm and left bundle branch block a significant improvement in NYHA functional class by 40%, exercise tolerance (6 minute walk distance by 30 % and peak V0_2_ by 26 %) and norepinephrine level by 37% [19]. Again and in agreement with the previous studies, a similar improvement in functional class and exercise performance was found in 2 groups of patients (BIV: 12 pts - LV: 14 pts) after 6 months follow-up in a non-randomised study, although, as expected, a significant decrease in QRS duration (-19 ms) was showed only in the BIV pacing group [17].

## LV pacing and ventricular asynchrony

Are these clinical effects correlated with improvement in myocardial synchrony? This is an important issue on a physiopathological point of view and for the selection of candidates for LV pacing. In an interesting experimental study using a canine model of cardiac failure and left bundle branch block, Leclercq et al examined mechanical and electrical synchrony during right atrial, LV and BIV stimulation; as expected, electrical dispersion decreased during BIV pacing whereas it increased during LV pacing; however, despite this opposite action upon electrical phenomena, the same improvement in mechanical coordination within the LV, measured by tagged magnetic resonance imaging, was observed with LV and BIV pacing, and this was correlated with improvement in hemodynamics (25 % increase in dP/dt and aortic pulse pressure) [7]. Similarly, in humans, LV dyssynchrony measured either by echocardiographic phase analysis, echo-contrast, or tissue Doppler imaging was improved significantly and almost at the same extent during LV and BIV pacing [16,21,22]. The decrease in the septal to lateral contraction delay during LV pacing was particularly correlated with an increase in LV ejection fraction or dP/dt [21,22] .

## Cost effectiveness

By comparison to BIV pacing, LV pacing offers several technical and economical advantages : 1) if insertion of a third lead (the right ventricular one) is not the most difficult part of the operative procedure, it could prolong significantly its duration and the X-ray exposure; 2) the presence of 3 leads instead  of 2 induces certainly more adverse events and increases the risk of venous thrombosis or mechanical complications; 3) more importantly, uniLV pacing needs only a less complex to program and less often subject to dysfunction conventional dual-chamber  pacemaker. Finally, BIV pacing is more expensive than LV pacing as it implies implantation of a supplementary right ventricular lead and of a specific and more costly device: in Europe, the extra cost could be approximately evaluated at 30 % and this must be taken in account in the context of the growing “epidemic” of heart  failure consecutive to demographic ageing and of the control of medical expenses in many countries.

So, at this time, there are no arguments in favour of the hypothesis that, for patients with severe CHF and enlarged QRS duration, BIV pacing would be significantly more effective than LV pacing in terms of mechanical coordination, hemodynamic and clinical benefits. However, our knowledge about the effects of LV pacing still suffers some limitations: the present studies are only observational and non-randomised. Furthermore, it seems, as shown during acute hemodynamic studies, that in some patients LV pacing would not be as effective as BIV pacing (but the opposite is true also… ), and this could perhaps be the case in patients with a dysfunctional right venricle but no study have focused on this topic at this time. Finally  and unlike BIV pacing [3,23], no study have examined the effects of LV pacing on mortality and morbidity, and whether or not LV pacing improves mortality in severe CHF remains to be demonstrated. However, the ongoing trials comparing LV to BIV pacing are not designed to show that one of these pacing modalities is better than the other but just equivalent… an argument to think that they are really similar.
